# Host nasopharyngeal transcriptome dataset of a SARS-CoV-2 positive Italian cohort

**DOI:** 10.1038/s41597-023-02289-7

**Published:** 2023-06-14

**Authors:** Annamaria Salvati, Carlo Ferravante, Jessica Lamberti, Teresa Rocco, Elena Alexandrova, Ylenia D’Agostino, Maksim Sorokin, Victor Efimov, Anton Buzdin, Oriana Strianese, Giovanni Nassa, Roberta Tarallo, Alessandro Weisz, Francesca Rizzo, Giorgio Giurato

**Affiliations:** 1 Molecular Pathology and Medical Genomics Program, Division of Oncology, AOU ‘S. Giovanni di Dio e Ruggi 14 d’Aragona’, Università di Salerno, Salerno, 84131 Italy; 2 Laboratory of Molecular Medicine and Genomics, Department of Medicine, Surgery and Dentistry ‘Scuola Medica Salernitana’, University of Salerno, Baronissi (Sa), 84081 Italy; 3 Moscow Institute of Physics and Technology, Dolgoprudny, Moscow Region 141701 Russia; 4 OmicsWay Corp, Walnut, USA; 5 Oncobox Ltd., Moscow, Russia; 6 World-Class Research Center ‘Digital biodesign and personalized healthcare’, Sechenov First Moscow State Medical University, Moscow, Russia; 7 Shemyakin-Ovchinnikov Institute of Bioorganic Chemistry, Moscow, 117997 Russia; 8 Genome Research Center for Health, Campus of Medicine, University of Salerno, Baronissi (Sa), 84081 Italy

**Keywords:** Gene expression profiling, SARS-CoV-2

## Abstract

The ongoing COVID-19 pandemic caused by SARS-CoV-2 has affected millions of people worldwide and has significant implications for public health. Host transcriptomics profiling provides comprehensive understanding of how the virus interacts with host cells and how the host responds to the virus. COVID-19 disease alters the host transcriptome, affecting cellular pathways and key molecular functions. To contribute to the global effort to understand the virus’s effect on host cell transcriptome, we have generated a dataset from nasopharyngeal swabs of 35 individuals infected with SARS-CoV-2 from the Campania region in Italy during the three outbreaks, with different clinical conditions. This dataset will help to elucidate the complex interactions among genes and can be useful in the development of effective therapeutic pathways.

## Background & Summary

Severe acute respiratory syndrome coronavirus 2 (SARS-CoV-2), responsible for coronavirus disease 19 (COVID-19), has emerged in December 2019 when the first case was reported in Wuhan, China. Soon after, it has rapidly spread to other countries worldwide, becoming pandemic with more than 4 million fatalities and 230 million cases registered^1^.

There are different factors that make it difficult to contain the spread of COVID-19. These include the high mutation rate of the virus, the challenge of diagnosing asymptomatic or mildly symptomatic individuals and the capability of the virus to be transmitted during the pre-symptomatic phase^2^.

After transmission processes through respiratory droplets, aerosol or surface contamination, follows the incubation period that could led to a plethora of symptoms such as fever, cough, shortness of breath, loss of taste and smell, diarrhea and nausea^3^. Nevertheless, a notable proportion of individuals with pre-existing conditions, such as asthma, diabetes, cardiovascular disease and other chronic illnesses experienced severe complications such as pneumonia affections or acute respiratory syndrome^4^. Some respiratory failures in severe SARS-CoV-2 infection have been found to be associated with the activation of immune response and pro-inflammatory mechanisms by chemokines and cytokine release, which may be caused by a “cytokine storm syndrome”^5^. In addition to pre-existing clinical conditions, other factors, such as age, sex, and ethnicity can also impact the clinical presentation of infected patients^6^.

As the vast range of disease susceptibility and outcomes observed in individuals infected with SARS-CoV-2 may be attributed to gene expression modulation resulting from virus-host cell interactions, several studies have been performed to investigate the biological effects of virus infection on the host transcriptome profile^7^. SARS-CoV-2 enters into the host cell by direct attachment to multiple receptors on the cell membrane or through membrane fusion within the endosome after endocytosis leading to further factors in human gene expression modulation^8^. SARS-CoV-2 primarily enters host cells through the angiotensin converting enzyme-2 (ACE2) located on the surface of different cell types. This interaction activates the renin-angiotensin pathway, which may increase the risk of severe COVID-19 symptoms in affected individuals^9^. Hence, upon detection of infection, human cells activate mechanisms to counteract viral replication which involves significant reprogramming of their own transcriptome^10^. Despite the worldwide spread, the host immune response against SARS-CoV-2 infection remains poorly characterized. Identifying transcriptome differences can be valuable for the determination of the cellular pathways that are modulated by the virus in infected cells.

**Fig. 1:**
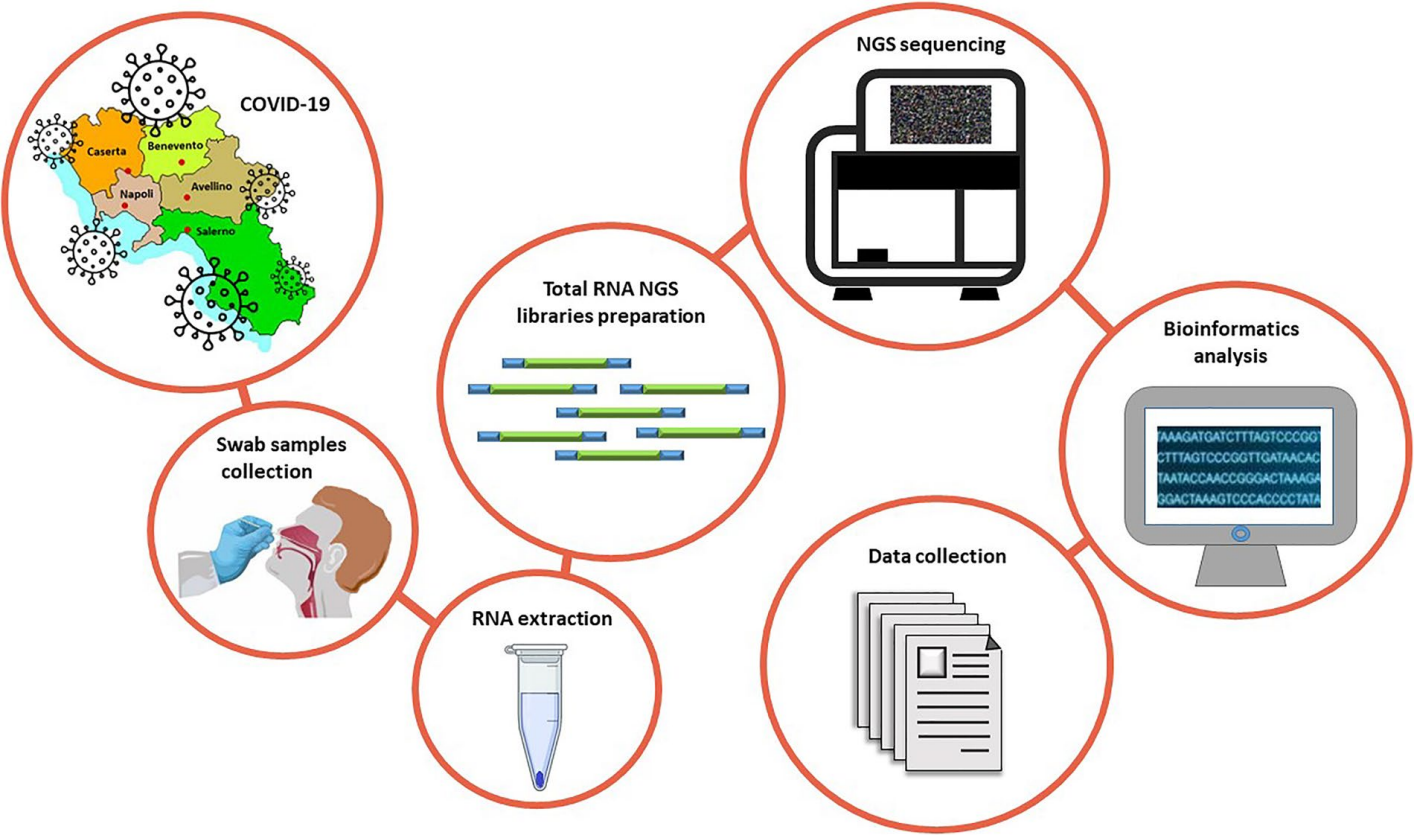
Experimental workflow. Summary of the experimental workflow applied to generate transcriptomic datasets originated by RNA extracted from nasopharyngeal swabs in the Campania cohort.

Here, our objective is to provide a comprehensive transcriptomic dataset of a cohort of SARS-CoV-2 positive Italian individuals. This dataset will allow the scientific community to study the impact of virus infection on the transcriptome of mucosa cells. To this aim, RNA extracted from 35 nasopharyngeal swabs of COVID-19 patients enrolled in the Campania region was subjected to total RNA sequencing and subsequent bioinformatics analysis (Fig. 1).

**Fig. 2:**
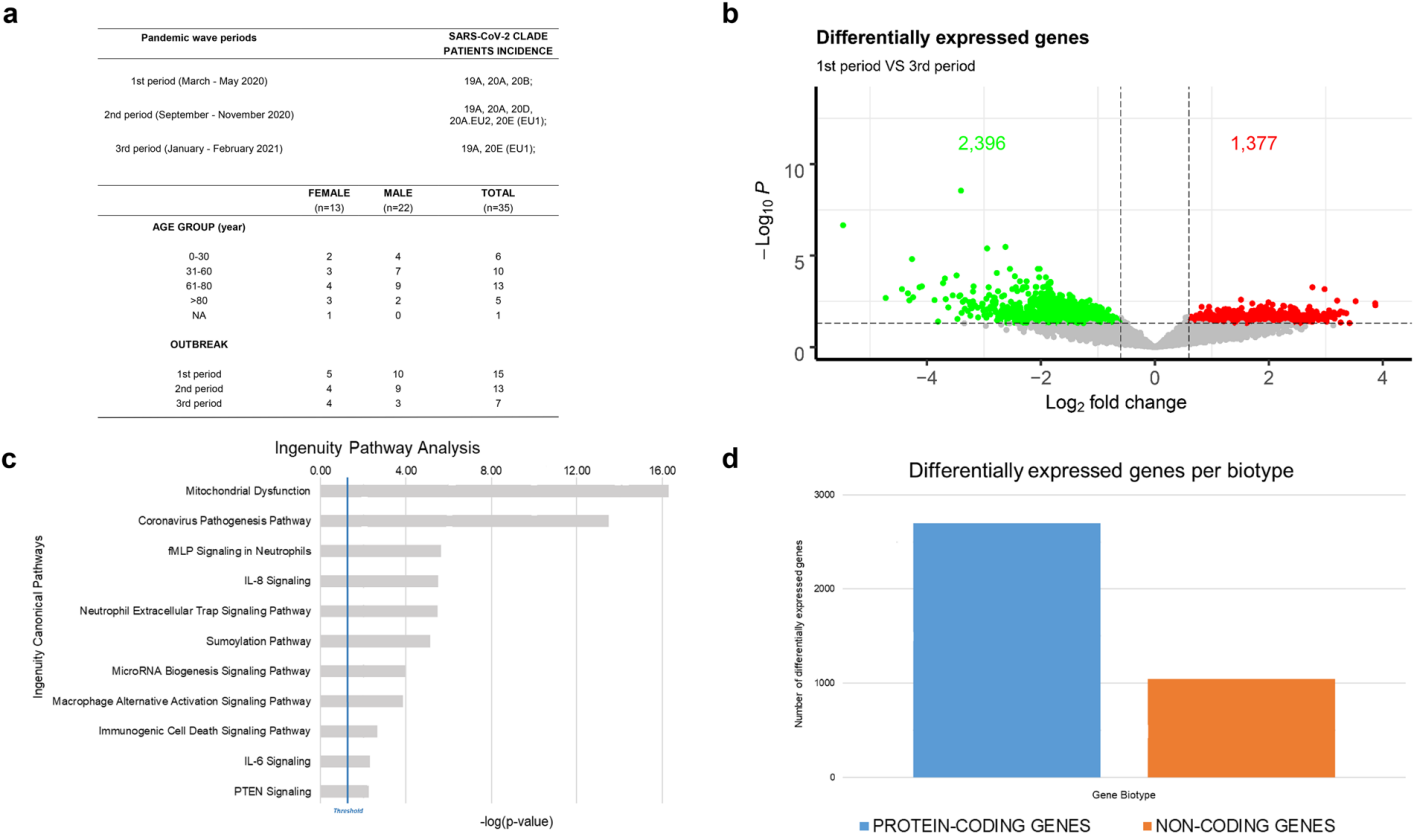
Analysis of RNA gene expression profiles between 1st and 3rd wave (**a**) Summary of demographics and other patients’ features of recruited cases in this cohort. (**b**) Volcano plot summarizing transcripts changes comparing 1st vs 3rd period. Green Dot and Red Dot show down- and up-regulated genes, respectively. According to the adjusted p-values (FDR) threshold of 0.05, transcripts associated with insignificant expression values are reported in grey. The dotted line (threshold) represents the cut-off (p-value ≤ 0.01). (**c**) Bar chart showing statistically significant pathway, according to IPA, of differentially expressed genes between 1st vs 3rd pandemic waves in Campania. (**d**) Bar chart showing the differentially expressed genes biotype detected in the analysis, divided into protein-coding (blue) and non-coding (orange) genes.

Patients were selected according to age, sex, sampling time and clinical manifestation of the disease (Fig. 2a and Supplementary File 1). Our sampling also covers the timing of the three different waves of SARS-CoV-2 infections in Italy, ranging from the pandemic declaration in March 2020 to spring 2021^11^. In detail, 15 cases belong to the 1st period (March-May 2020), 13 to the 2nd period (September - November 2020) and 7 to the 3rd period (January - February 2021) (Fig. 2a).

Interestingly, by total RNA approach and deep sequencing conditions, detailed in the Methods section, our bioinformatics analyses have detected also reads aligned on the SARS-CoV-2 genome. In this way, we are also able to observe the distribution of virus variants peculiar to different pandemic waves, which may contribute to host response variability analysis (Fig. 2a and Supplementary File 1).

The transcriptome dataset here proposed, can provide valuable insights into the biological impact of SARS-CoV-2 infection on the modulation of host gene expression. By analyzing this dataset and integrating it with others, researchers could identify key protein-coding and non-coding genes involved in pathways affected by the virus’s entrance. This could help in the development of new therapies and diagnostic tools.

Moreover, this dataset includes several clinical factors which can be used to study the relationship between these factors and the host’s gene expression changes induced by SARS-Cov-2 infection.

Additionally, the clade assignment provides an opportunity to investigate the potential differences in transcriptome profiles between different viral strains. This can help in understanding the pathogenesis of the disease and the potential differences in virulence and transmissibility among different SARS-CoV-2 variants. Overall, the transcriptome dataset from the Italian cohort of these patients is a valuable resource for researchers to be integrated with other datasets and identify potential therapeutic targets and diagnostic biomarkers.

## Methods

### Cohort and clinical samples

The cohort analyzed in our study includes 35 patients from Campania region hospitals in Italy, selected after confirmed SARS-CoV-2 infection by PCR testing in the nasopharyngeal swab. The study was approved by the Campania Sud Ethics Committee (approval code 206/2021) and was conducted according to the guidelines of the Declaration of Helsinki. The individuals consented to their data being published under an open license.

### RNA samples collection and quality controls

RNA purity was determined by using NanoDrop spectrophotometer ND-2000 (Thermo Fischer Scientific) through the evaluation of the absorbance ratio A260/A280. Total RNAs concentration was measured using RNA HS kit on a Qubit fluorimeter (ThermoFisher Scientific) while their integrity was evaluated by TapeStation 2200 (Agilent). The total RNA for each sample was reverse transcribed to cDNA using Random Hexamer (Tetro cDNA Synthesis Kit, Bioline, Memphis, Tennessee). RT-qPCRs were carried out by SensiFAST SYBR Lo-ROX kit (Bioline), according to the manufacturer’s instructions, targeting the viral N gene by following primers:

*Forward primers- GGGGAACTTCTCCTGCTAGAAT*

*Reverse primers-CAGACATTTTGCTCTCAAGCTG*

### RNA Seq library preparation and sequencing

100 ng of each RNA sample was used for sequencing library preparation using the TruSeq Stranded Total RNA Sample Prep kit protocol (Illumina Inc., San Diego, CA, USA). Then, 33 libraries were equimolarly pooled, diluted to a final concentration of 1.2 nMol and sequenced on NovaSeq 6000 (Illumina Inc) in a paired-end mode (2 × 100 base pairs). The 2 remaining libraries were diluted to a final concentration of 1.7 pMol and sequenced on NextSeq 500 (Illumina Inc) in a paired-end mode (2 × 75 base pairs).

### Bioinformatics and functional annotation analyses

After sequencing, the demultiplexing step was performed using Illumina bcl2fastq software, in order to generate FASTQ files for the subsequent analysis. Raw FASTQ files were quality checked using FastQC (v0.11.8)^12^ and trimmed with Cutadapt software (v4.2)^13^ using the following parameters:–minimum-length/-m and–quality-cutoff/-q options set as 20 and 25 respectively. The read alignment on the human reference genome (GRCh38/hg38) was performed using STAR software (v 2.7.4a)^14^ with default parameters and gene quantification was obtained with FeatureCounts v2.0.0^15^. The counts were then imported in R (version 3.6.3) and differentially expressed genes were identified using R package DESeq 2 v1.26.0^16^. Differential expression was reported as |fold change| ≥ 1.5 along with associated adjusted p-value (false discovery rate (FDR)) ≤ 0.05, computed according to Benjamini–Hochberg^17^, as described in Salvati *et al*.^18^. Functional analysis has been performed using IPA (Ingenuity Pathway Analysis). Only functions and pathways showing a p-value ≤ 0.05 have been considered. In addition, for the identification of SARS-CoV-2 virus strains, the reads that did not map on the human reference genome, were aligned on the SARS-CoV-2 genome (primary assembly MN908947.3) using Burrows–Wheeler Aligner (BWA) software (v0.7.17)^19^. Nucleotide variants were identified using Freebayes v.1.0.2^20^ and clade assignment was performed using Nextclade (https://clades.nextstrain.org/).

## Data Records

Complete RNA-seq data were deposited in the ArrayExpress database (https://www.ebi.ac.uk/biostudies/arrayexpress) under the accession number E-MTAB-13028^21^. Other data, such as list of differentially expressed genes, raw gene counts, R code used for differential expression analysis and list of canonical pathways are accessible through Figshare platform^22^.

## Technical Validation

**Fig. 3:**
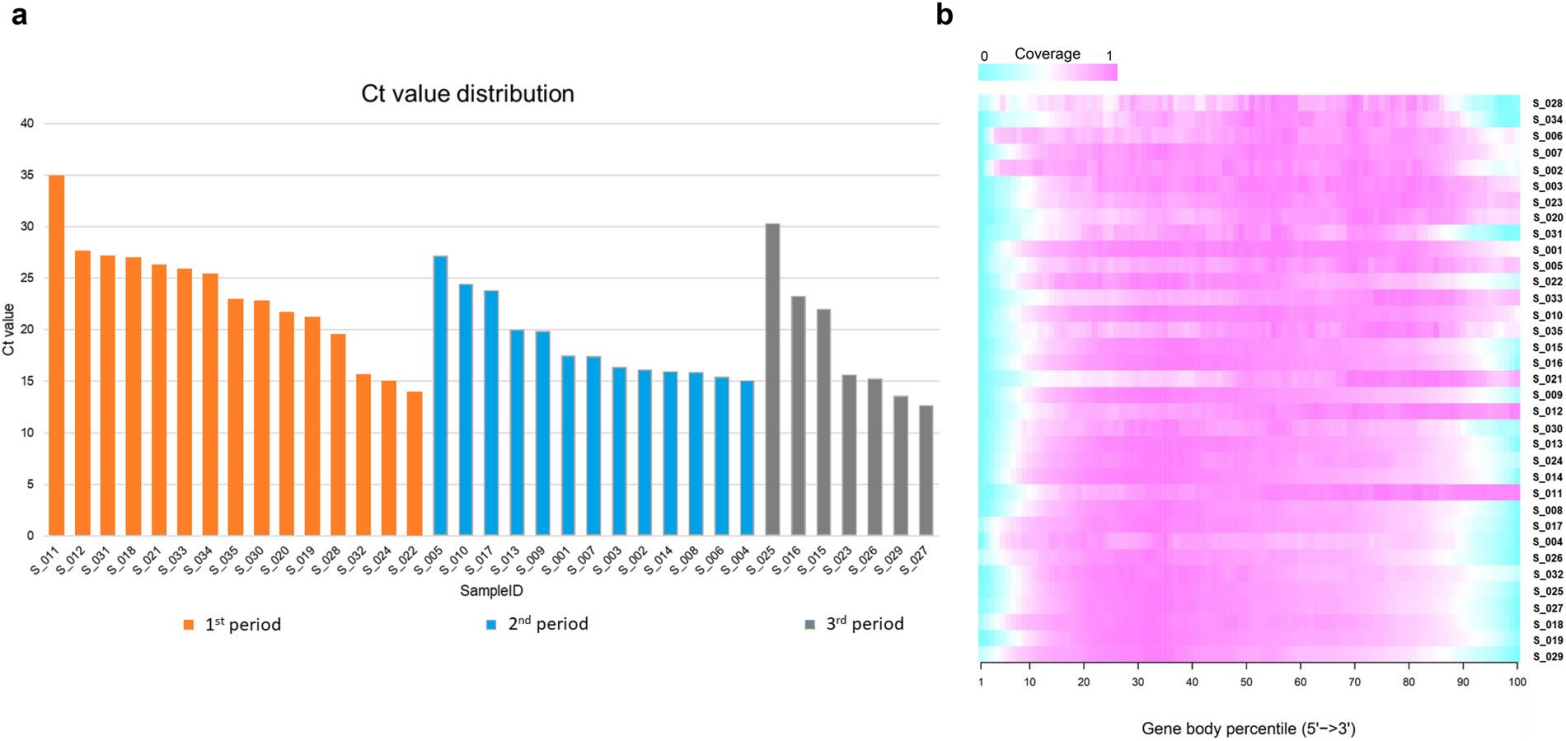
Quality controls of the experimental procedure. (**a**) Bar chart showing the Ct value associated with each patient distributed in three different waves (orange: 1st period, blue: 2nd period, grey: 3rd period. (**b**) Coverage plot showing the reads coverage along gene body. All transcripts for all samples were scaled to 100 nt length and the number of reads covering each nucleotide position was reported as coverage ranged from 0 (jade-green) to 1 (pink).

To verify the quality and robustness of the data presented here, cycle threshold (Ct) values were also reported (Fig. 3a) based on primers amplification of conserved viral genome regions by Reverse transcription-quantitative polymerase chain reaction (RT-qPCR) to rapidly track viral copies numbers in RNA extracted from swabs of all 35 patients^23^ (Fig. 3a and Supplementary file 2). In general, these specimens harbor high Ct values corresponding to low virus load and vice versa. Several studies aimed to detect the relationship between Ct and infectious virus activity but could be an imperfect measurement associated with laboratory-dependent procedures^24^. In this context, allowed a rapid method to confirm COVID-19-infected patients, highlighting the uniform distribution of high and low Ct values in each period (Fig. 3a). To evaluate the quality of the RNA-Seq data produced, the coverage along gene body was assessed and reported in Fig. 3b.

The same RNAs were then used for total RNA library preparation and sequencing, as described in the Methods section. The sequencing step produced 3,494,055,062 reads, with an average of 99,830,145 reads per sample (range 26,853,188–197,633,680). After trimming of low-quality and adapter related fragments, a total of 36,847,520 reads were removed from the dataset, corresponding to 1,052,786 reads per sample (range 45,944–2,592,362). The high-quality reads are then mapped to the human reference genome resulting in 73.11% of read mapping per sample.

Since the major clinical feature of COVID-19 is a hyperinflammatory state, characterized by high expression of cytokines and chemokines, as further technical validation of our dataset we investigated if these pathways were affected. This feature was analysed performing a differential gene expression analysis comparing patients of the first and the third period (Fig. 2b), due to the variations in COVID-19 restrictions implemented during the three distinct periods and the circulation of different viruses’ variants. The analysis highlighted 1,345 up- and 2,396 down-regulated transcripts (Figshare File 1 and File 2^22^). Functional analysis, performed with Ingenuity pathway analysis (IPA), confirmed the strong impact of COVID-19 infection in the modulation of target transcripts in host cells (Fig. 2c) and revealed their involvement in several biological mechanisms associated with immune cell response and coronavirus pathogenesis pathway^7,25^ (Figshare File 4 and File 5^22^).

Concerning differentially expressed genes, as expected, the majority of them are protein-coding (2,729 genes), while, interestingly a huge part (1,044) belong to the biotype class of non-coding RNA (ncRNA) (Fig. 2d). This aspect suggests how these data could be integrated with other datasets or other information to provide a detailed picture of SARS-CoV-2 infection, taking in consideration the known key role of ncRNAs into the mechanisms driving severe COVID-19^26^.

Analysis with covariates, such as age, sex and diseases status resulted in 250 differentially expressed genes. The observed result may be attributed to the limited number of patients when stratified based on these characteristics. In fact, the limitation of this particular dataset could be the small sample size, which may limit the statistical power of the analysis. However, the strength of the dataset lies in the accuracy and variety of patient features reported, which could provide valuable insight into the impact of COVID-19 on gene expression mechanisms.

The integration of this dataset with similar ones, along with a rigorous data validation process, could help to increase the statistical power and reliability of the findings.

## Supplementary information

Supplementary Table S2

Supplementary Table S1

## Data Availability

The R code used to perform differential expression analysis is available in FigShare File 3^22^.
