# Author Correction: Host nasopharyngeal transcriptome dataset of a SARS-CoV-2 positive Italian cohort

**DOI:** 10.1038/s41597-023-02504-5

**Published:** 2023-09-07

**Authors:** Annamaria Salvati, Carlo Ferravante, Jessica Lamberti, Teresa Rocco, Elena Alexandrova, Ylenia D’Agostino, Maksim Sorokin, Victor Efimov, Anton Buzdin, Oriana Strianese, Giovanni Nassa, Roberta Tarallo, Alessandro Weisz, Francesca Rizzo, Giorgio Giurato

**Affiliations:** 1 Molecular Pathology and Medical Genomics Program, Division of Oncology, AOU ‘S. Giovanni di Dio e Ruggi 14 d’Aragona’, Università di Salerno, Salerno, 84131 Italy; 2 Laboratory of Molecular Medicine and Genomics, Department of Medicine, Surgery and Dentistry ‘Scuola Medica Salernitana’, University of Salerno, Baronissi (Sa), 84081 Italy; 3 Moscow Institute of Physics and Technology, Dolgoprudny, Moscow Region 141701 Russia; 4 OmicsWay Corp, Walnut, USA; 5 Oncobox Ltd., Moscow, Russia; 6 World-Class Research Center ‘Digital biodesign and personalized healthcare’, Sechenov First Moscow State Medical University, Moscow, Russia; 7 Shemyakin-Ovchinnikov Institute of Bioorganic Chemistry, Moscow, 117997 Russia; 8 Genome Research Center for Health, Campus of Medicine, University of Salerno, Baronissi (Sa), 84081 Italy

**Keywords:** Gene expression profiling, SARS-CoV-2

Correction to: *Scientific Data* 10.1038/s41597-023-02289-7, published online 14 June 2023

In the Acknowledgements section of this article the grant number relating to World-Class Research Center ‘Digital biodesign and personalized healthcare’ given for Anton Buzdin and Maksim Sorokin was incorrectly given as No 075-15-2020-926 and should have been No 075-15-2022-304. The original article has been corrected.

